# Reactivation of wild-type and mutant p53 by tryptophanolderived oxazoloisoindolinone SLMP53-1, a novel anticancer small-molecule

**DOI:** 10.18632/oncotarget.6775

**Published:** 2015-12-28

**Authors:** Joana Soares, Liliana Raimundo, Nuno A.L. Pereira, Ângelo Monteiro, Sara Gomes, Cláudia Bessa, Clara Pereira, Glória Queiroz, Alessandra Bisio, João Fernandes, Célia Gomes, Flávio Reis, Jorge Gonçalves, Alberto Inga, Maria M.M. Santos, Lucília Saraiva

**Affiliations:** ^1^ UCIBIO/REQUIMTE, Universidade do Porto, Porto, Portugal; ^2^ Laboratório de Microbiologia, Departamento de Ciências Biológicas, Faculdade de Farmácia, Universidade do Porto, Porto, Portugal; ^3^ Laboratório de Farmacologia, Departamento de Ciências do Medicamento, Faculdade de Farmácia, Universidade do Porto, Porto, Portugal; ^4^ Research Institute for Medicines (iMed.ULisboa), Faculty of Pharmacy, Universidade de Lisboa, Lisboa, Portugal; ^5^ CIBIO, Centre for Integrative Biology, Laboratory of Transcriptional Networks, University of Trento, Trento, Italy; ^6^ Laboratório de Farmacologia e Terapêutica Experimental, IBILI, Faculdade de Medicina, Universidade de Coimbra, Coimbra, Portugal

**Keywords:** tumor, p53, mutant, tryptophanol-derived oxazoloisoindolinones, anticancer chemotherapy

## Abstract

Restoration of the p53 pathway, namely by reactivation of mutant (mut) p53, represents a valuable anticancer strategy. Herein, we report the identification of the enantiopure tryptophanol-derived oxazoloisoindolinone SLMP53-1 as a novel reactivator of wild-type (wt) and mut p53, using a yeast-based screening strategy. SLMP53-1 has a p53-dependent anti-proliferative activity in human wt and mut p53R280K-expressing tumor cells. Additionally, SLMP53-1 enhances p53 transcriptional activity and restores wt-like DNA binding ability to mut p53R280K. In wt/mut p53-expressing tumor cells, SLMP53-1 triggers p53 transcription-dependent and mitochondrial apoptotic pathways involving BAX, and wt/mut p53 mitochondrial translocation. SLMP53-1 inhibits the migration of wt/mut p53-expressing tumor cells, and it shows promising p53-dependent synergistic effects with conventional chemotherapeutics. In xenograft mice models, SLMP53-1 inhibits the growth of wt/mut p53-expressing tumors, but not of p53-null tumors, without apparent toxicity. Collectively, besides the potential use of SLMP53-1 as anticancer drug, the tryptophanol-derived oxazoloisoindolinone scaffold represents a promissing starting point for the development of effective p53-reactivating drugs.

## INTRODUCTION

The p53 transcription factor finely regulates the expression of genes with central roles in cellular processes including DNA repair, cell cycle, and apoptosis [1, 2]. Compelling evidence has demonstrated that inactivation of the p53 pathway is required for tumor development and maintenance. In fact, in all human cancers bearing p53, this protein is inactivated by binding to endogenous inhibitors, such as murine double minute (MDM)2, or by mutation of the *TP53* gene [1, 2]. Therefore, p53 reactivation represents a highly effective strategy in cancer treatment [1].

The majority of p53 mutations are missense, preferentially localized in the DNA binding domain. They can be classified as structural (e.g., R175H, Y220C) or DNA contact (e.g., R273H, R280K) based on whether or not they lead to a profound protein conformational change, respectively. However, both types of mutations prevent the p53 binding to DNA, leading to the loss of wild-type (wt) p53 transcriptional activity [1–4]. Additionally, *TP53* mutations may lead to the acquisition of gain-of-function (GOF) activities, responsible for more aggressive tumor phenotypes with increased invasive and metastatic potential, high chemoresistance, and poor prognosis [3–5]. Moreover, high levels of mutant (mut) p53 are often found in tumors due to a reduced MDM2 expression, and consequent impairment of p53 degradation [3]. Collectively, these observations support that mut p53 is a promising therapeutic target against a wide range of aggressive tumors [3–5].

To date, few small-molecule reactivators of mut p53 have been identified. Actually, due to its structural nature, the restoration of wt-like function to mut p53 has been challenging. Moreover, for most of the reported mut p53 reactivators, unfavorable pharmacokinetics and toxicity profiles have been described [6]. In fact, so far, only the PRIMA-1 derivative, APR-246, has reached clinical trials [1–3]. Therefore, more pharmacological alternatives for mut p53 reactivation are still required.

**Figure 1:**
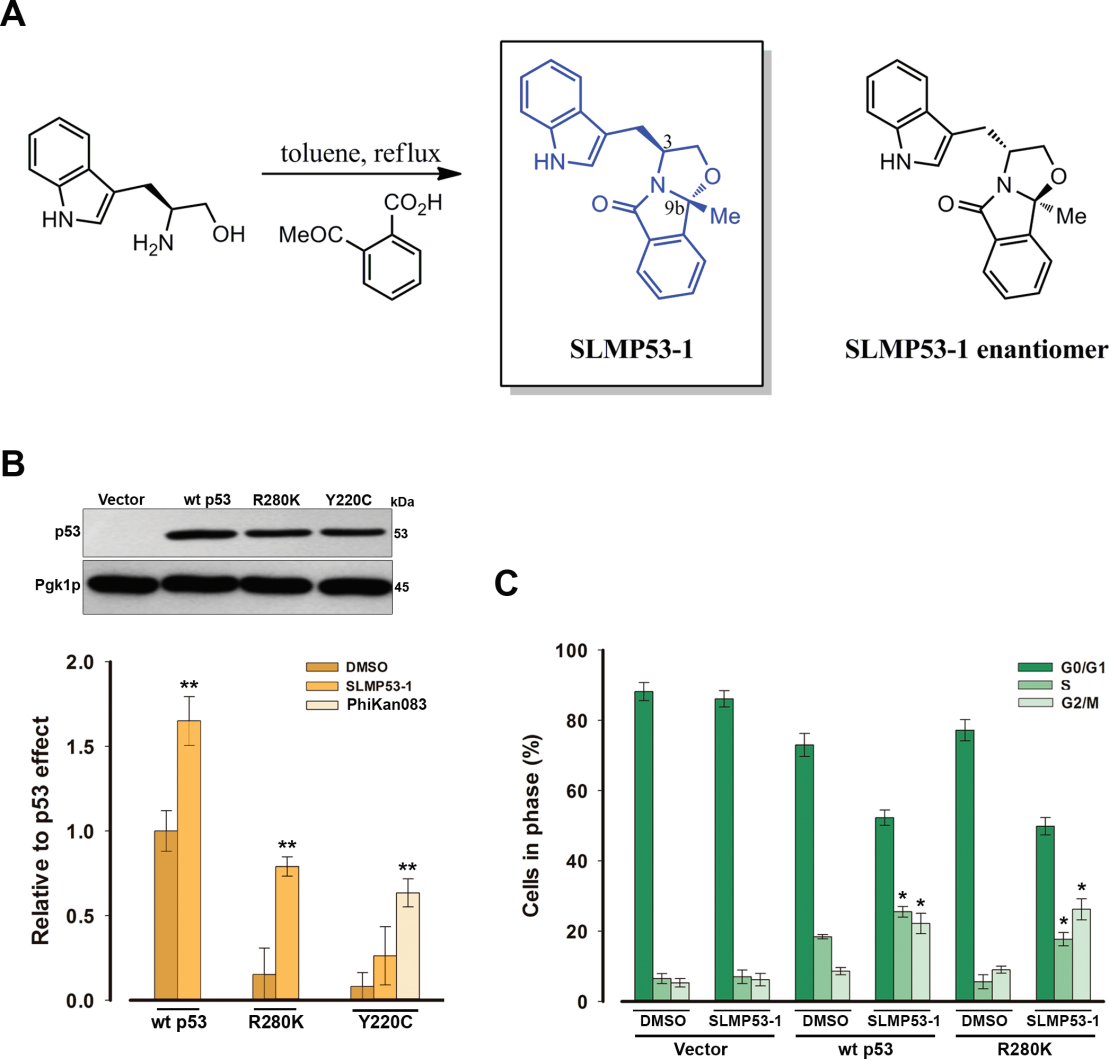
SLMP53-1 activates wt p53 and restores the wt-like activity to mut p53R280K in yeast (**A**) Synthesis of SLMP53-1 and chemical structure of its enantiomer. (**B**) Immunoblots of human wt p53, mut p53R280K and mut p53Y220C expressed in yeast after 30 h incubation in selective induction medium (representative of three independent experiments). Graphical representation: increase of wt and mut p53R280K-induced growth inhibition by 10 μM SLMP53-1 and of mut p53Y220C-induced growth inhibition by 50 μM PhiKan083, after 30 h; results were plotted setting as 1 the growth inhibitory effect of wt p53-expressing cells treated with DMSO only; data are mean ± SEM (*n* = 5). (**C**) Increase of wt and mut p53R280K-induced yeast cell cycle arrest by 10 μM SLMP53-1; data are mean ± SEM (*n* = 3). In B and C, values significantly different from DMSO only: *p < 0.05, **p < 0.01.

In this work, the enantiopure tryptophanol-derived oxazoloisoindolinone SLMP53-1 (Figure 1A) was identified as a new reactivator of wt and mut p53R280K. This compound revealed *in vitro* and *in vivo* p53-dependent antitumor activity, both alone and combined with conventional chemotherapeutics, against tumors bearing wt/mut p53.

## RESULTS

### Identification of SLMP53-1 as activator of wt p53 and reactivator of mut p53R280K from a yeast screening of a library of tryptophanol-derived oxazoloisoindolinones

A yeast-based screening assay, consisting of *Saccharomyces cerevisiae* cells expressing human wt p53 or mut p53 (R280K or Y220C; two of the most prevalent forms of human mut p53 [7]), was developed to search for mut p53 reactivators (Figure 1B). This assay was established based on the yeast growth inhibitory effect induced by wt p53, but not by mut p53 [8]. Based on this phenotypic readout, mut p53 reactivators would reestablish the wt p53-dependent growth inhibition. Since, to date, mut p53R280K reactivators remain unknown, the mut p53Y220C reactivator PhiKan083 [9] was used to attest the efficacy of the yeast assay (Figure 1B). In yeast, 50 μM PhiKan083 restored the wt p53-induced growth inhibition to mut p53Y220C (in about 63%; Figure 1B).

This yeast assay was thereafter used to evaluate the effect of synthesized enantiopure tryptophanol-derived oxazoloisoindolinones on wt and mut p53 activity. These compounds were synthesized following our interest in enantiopure biologically active amino alcohol-derived compounds [10–12], and on our previous discovery of phenylalaninol-derived oxazoloisoindolinones with *in vitro* p53-dependent antitumor activity [10]. With this new library, we intended to study the effect on p53 activity of the replacement of the phenyl moiety (present in our first set of compounds [10]) by an indole moiety. Among the compounds tested, SLMP53-1 behaved as an activator of wt p53 and reactivator of mut p53R280K (Figure 1B and 1C). Actually, at 10 μM (lowest concentration, from a range of 1–50 μM, that caused a maximal effect; data not shown), SLMP53-1 increased the wt p53-induced yeast growth inhibition and restored the wt-like growth inhibitory effect to mut p53R280K (in about 79%; Figure 1B), without interfering with the growth of control yeast (data not shown). Consistently, 10 μM SLMP53-1 increased the S- and G2/M-phases cell cycle arrest in wt p53-expressing yeast, and reestablished the wt-like cell cycle arrest in mut p53R280K-expressing yeast (Figure 1C). Interestingly, 1–50 μM SLMP53-1 did not interfere with the growth of mut p53Y220C-expressing yeast (Figure 1B).

A previous work [13] corroborated the conservation in yeast of the p53 transcriptional activity, and established its correlation with p53-induced growth inhibition using pifithrin-α (PFT-α; known selective inhibitor of p53 transcriptional activity [14]). Accordingly with previous results [13], in the present work, the treatment of yeast cells expressing wt p53 with 5 μM PFT-α reduced the wt p53-induced growth inhibition by 68.0 ± 1.1% (*n* = 4). Additionally, in the presence of 5 μM PFT-α, 10 μM SLMP53-1 increased the wt p53-inhibitory effect by only 5.8 ± 0.8% (*n* = 4; relative to PFT-α only). A similar result was obtained with yeast cells expressing mut p53R280K. In fact, in these cells, 10 μM SLMP53-1 induced a mut p53 inhibitory effect of 2.5 ± 1.1% (*n* = 4; relative to PFT-α only). These results established a correlation between the activation of p53 transcriptional activity by SLMP53-1 and its cytotoxicity in yeast, since in the presence of a p53 transcriptional inhibitor, the activity of SLMP53-1 was abolished.

### Growth inhibitory effect of SLMP53-1 on wt p53- and mut p53R280K-expressing tumor cells is mediated by a p53-dependent cell cycle arrest and/or apoptosis

**Figure 2:**
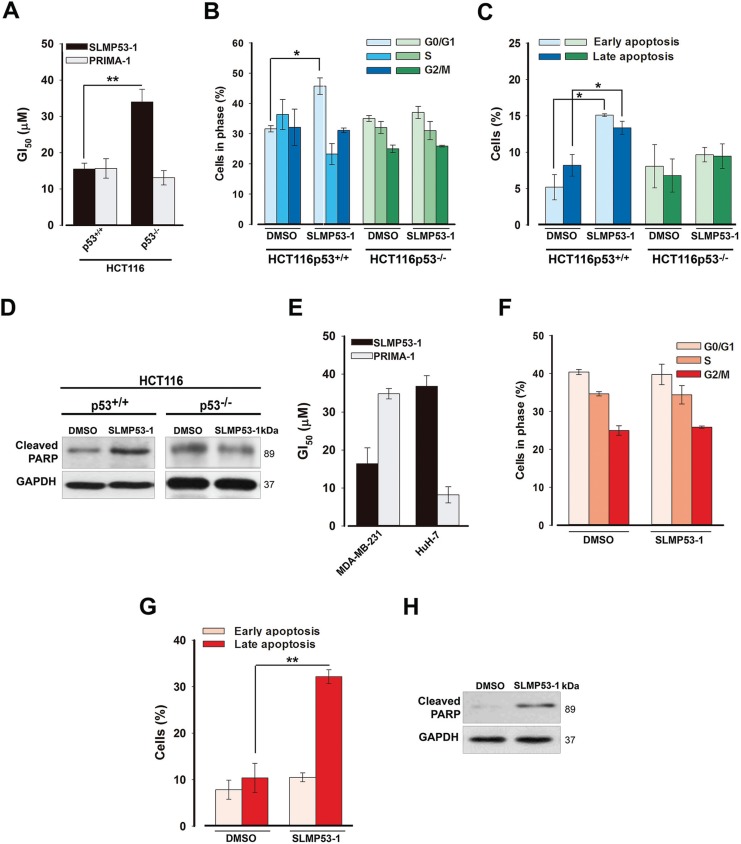
SLMP53-1 growth inhibitory effect in human wt p53- and mut p53R280K-expressing tumor cells is mediated by p53-dependent cell cycle arrest and/or apoptosis (**A**) GI_50_ value of SLMP53-1 in HCT116 cells, after 48 h treatment; values significantly different: **p < 0.01. (**B**) Cell cycle, (**C**) Apoptosis and (**D**) PARP cleavage after 24 h with 16 μM SLMP53-1 in HCT116 cells. (**E**) GI_50_ value for SLMP53-1 in MDA-MB-231 (mut p53R280K) and HuH-7 (mut p53Y220C) cells, after 48 h treatment. (**F**) Cell cycle, (**G**) Apoptosis and (**H**) PARP cleavage after 24 h with 16 μM SLMP53-1 in MDA-MB-231 cells. In A–C and E–G, data are mean ± SEM (*n* = 3–4). In A, B, C and G, values significantly different from DMSO only: *p < 0.05, **p < 0.01. In F, values are not significantly different from DMSO only: *p* > 0.05. In D and H immunoblots are representative of three independent experiments.

The growth inhibitory potential of SLMP53-1, and the contribution of the p53 pathway to its activity, were ascertained in p53^+/+^ and p53^−/−^ HCT116 tumor cells. A significant difference between the GI_50_ (concentration for 50% of maximal growth inhibition) values obtained in p53^+/+^ (~16 μM) and p53^−/−^ (~34 μM) HCT116 cells indicated an involvement of p53 in the growth inhibitory effect of SLMP53-1 (Figure 2A). Conversely, no significant differences were observed between the GI_50_ values of PRIMA-1 in p53^+/+^ and p53^−/−^ HCT116 cells (Figure 2A). In our experimental conditions, the GI_50_ value of PhiKan083 in HCT116 cells was higher than 150 μM.

The SLMP53-1 growth inhibitory effect was associated with G0/G1-phase cell cycle arrest (Figure 2B), and apoptosis (Figure 2C) in p53^+/+^, but not in p53^−/−^, HCT116 cells. The enhancement of PARP cleavage by 16 μM SLMP53-1 in p53^+/+^, but not in p53^−/−^, HCT116 cells further corroborated the activation of a p53-dependent apoptotic pathway (Figure 2D).

Consistent with the yeast results, SLMP53-1 also inhibited the growth of mut p53R280K-expressing MDA-MB-231 cells with a GI_50_ value similar to that obtained in HCT116p53^+/+^ cells (~16 μM; Figure 2E). Moreover, it presented a much lower growth inhibitory activity against mut p53Y220C-expressing HuH-7 cells (Figure 2E). When compared to PRIMA-1, SLMP53-1 presented a higher potency in MDA-MB-231 cells, but was less potent against mut p53Y220C-expressing HuH-7 cells (Figure 2E). In our experimental conditions, the GI_50_ values of PhiKan083 in MDA-MB-231 and HuH-7 cells were higher than 150 μM.

Interestingly, in MDA-MB-231 cells, despite not interfering with cell cycle progression (Figure 2F), 16 μM SLMP53-1 stimulated cell death, as revealed by the increase of late apoptosis (Figure 2G) and PARP cleavage (Figure 2H).

### SLMP53-1 selectively activates p53 transcriptional activity and reestablishes mut p53R280K DNA binding ability

The effect of SLMP53-1 on wt p53 and mut p53R280K activity was investigated by analysis of the expression levels of proteins encoded by p53 target genes in HCT116p53^+/+^, HCT116p53^−/−^, and MDA-MB-231 cells.

**Figure 3:**
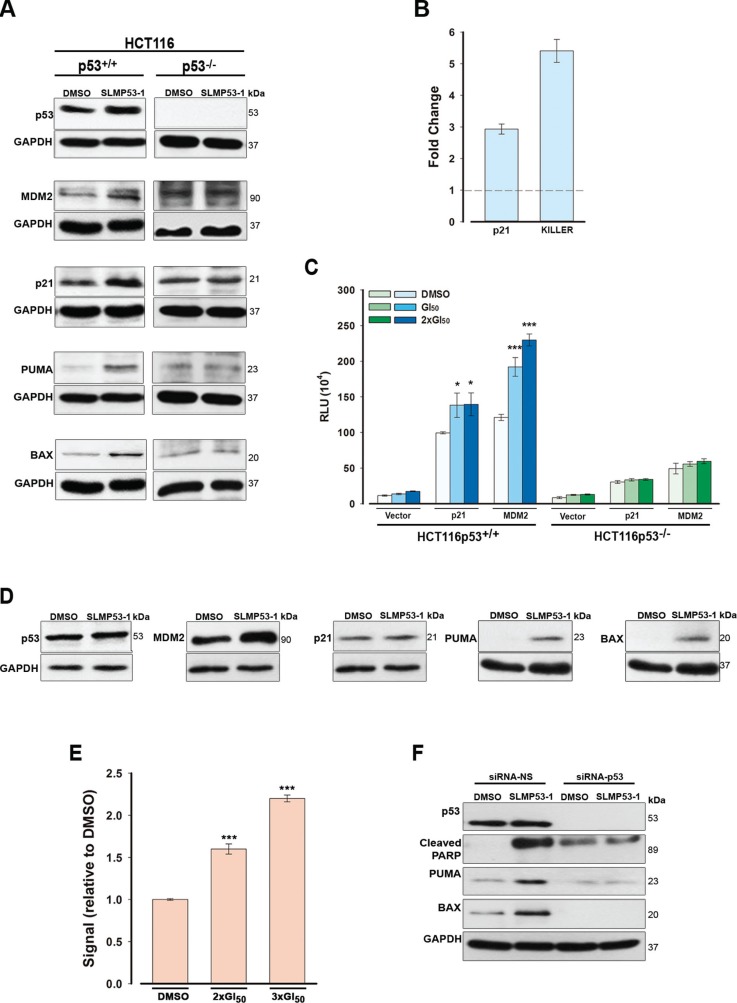
SLMP53-1 reactivates the transcriptional activity of wt p53 in HCT116p53^+/+^ cells and mut p53R280K, through reestablishment of its DNA binding ability, in MDA-MB-231 cells (**A**) Protein levels of p53 target genes, and (**B**) gene expression of *CDKN1A* (p21) and *TNFRSF10B* (KILLER), after 24 h with 16 μM SLMP53-1 in HCT116 cells. In B, fold expression changes are relative to DMSO and correspond to mean ± SEM (*n* = 3). (**C**) p53 transcriptional activity after 16 h with 16 μM and 32 μM (2 × GI_50_) SLMP53-1 in HCT116 cells assessed by dual-luciferase gene reporter assay. Data are plotted as mean relative light units (RLU) ± SEM (*n* = 3); values significantly different from DMSO only: *p < 0.05, ***p < 0.001. (**D**) Protein levels of p53 target genes after 24 h with 16 μM SLMP53-1 in MDA-MB-231 cells. (**E**) DNA binding affinity of mut p53R280K after 24 h with 32 μM and 48 μM (3 × GI_50_) SLMP53-1 in MDA-MB-231 cells; the signal obtained with DMSO only was set as 1; data are mean ± SEM (*n* = 3); values significantly different from DMSO only: ***p < 0.001. (**F**) PARP cleavage and protein levels of PUMA and BAX after 24 h with 16 μM SLMP53-1 in mut p53R280K-silenced MDA-MB-231 cells. In A, D and F, immunoblots are representative of three independent experiments.

In HCT116p53^+/+^ cells, 16 μM SLMP53-1 increased the p53, MDM2, p21, PUMA and BAX expression levels (Figure 3A). Instead, the levels of these proteins were not significantly changed in HCT116p53^−/−^ cells treated with 16 μM SLMP53-1, supporting the p53-dependent activity of this molecule (Figure 3A). Additionally, 16 μM SLMP53-1 increased the mRNA levels of *CDKN1A* (p21) and *TNFRSF10B* (KILLER) in HCT116p53^+/+^ cells (Figure 3B). The selective activation of p53 transcriptional activity by SLMP53-1 was further reinforced by a dual-luciferase gene reporter assay using p53^+/+^ and p53^−/−^ HCT116 cells transfected with p21- or MDM2-luciferase reporter vectors. At 16 μM and 32 μM (2 × GI_50_), SLMP53-1 increased the expression of the two p53 reporters in HCT116p53^+/+^ cells, while no effect was observed in HCT116p53^−/−^ cells (Figure 3C).

In MDA-MB-231 cells, 16 μM SLMP53-1 increased MDM2, PUMA and BAX protein levels, with no apparent increase of p53 protein levels (Figure 3D). The lack of mut p53 protein stabilization was already reported for other mut p53 reactivators, and has been attributed to a reestablishment of the MDM2-mediated p53 degradation [15]. Moreover, consistent with the lack of effect on cell cycle progression, 16 μM SLMP53-1 did not affect p21 protein levels (Figure 3D).

The restoration of mut p53R280K DNA binding ability by SLMP53-1 was assessed by TransAM DNA binding assay, using MDA-MB-231 cells. The results revealed a dose-dependent enhancement of the amount of p53 bound to DNA by SLMP53-1 relative to cells treated with DMSO only (Figure 3E).

To further demonstrate that the SLMP53-1 effects were dependent on mut p53R280K in MDA-MB-231 cells, mut p53R280K was knocked-down by p53siRNA. In cells transfected with non-specific siRNA (siRNA-NS; control), 16 μM SLMP53-1 increased the protein levels of PUMA, BAX and cleaved PARP, as observed in non-transfected cells (Figure 3F). As expected, these SLMP53-1 effects were efficiently abolished by mut p53 silencing (Figure 3F).

### SLMP53-1 triggers a p53-dependent mitochondrial apoptotic pathway in wt p53- and mut p53R280K-expressing tumor cells

**Figure 4:**
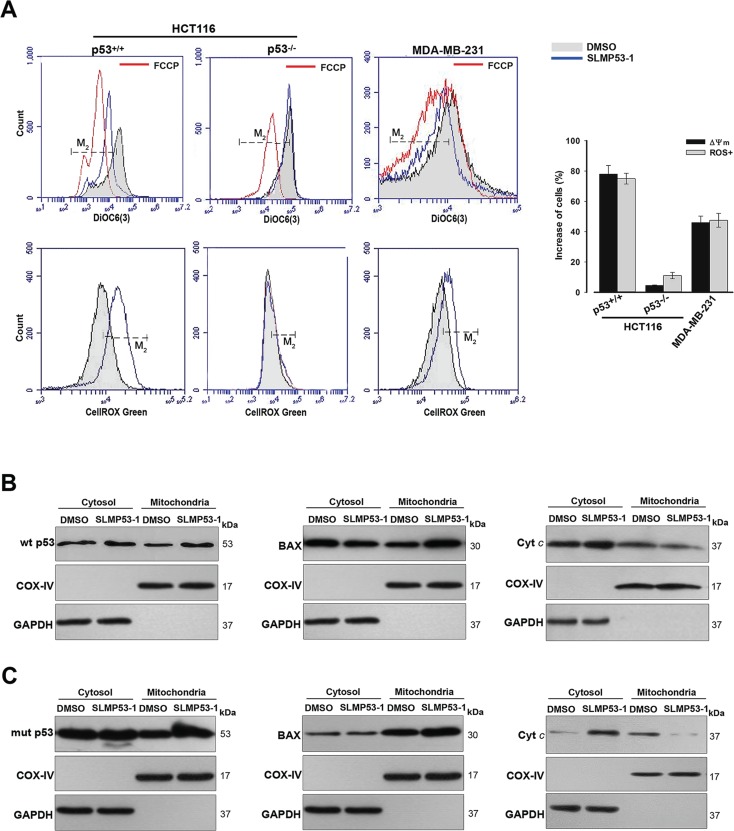
SLMP53-1 triggers a p53-mediated mitochondrial apoptotic pathway in HCT116p53^+/+^ and MDA-MB-231 cells (**A**) Cells were treated with 16 μM SLMP53-1 for 8 h (HCT116 cells) or 16 h (MDA-MB-231 cells) in Δψ_m_ analysis, and for 24 h in ROS analysis. Carbonyl cyanide-*p*-trifluoromethoxyphenylhydrazone (FCCP) was used as positive control in Δψ_m_; histograms are representative of three independent experiments; M_2_ cursor indicates the subpopulation analyzed. Graphical representation: increase in the percentage of cells with Δψ_m_ dissipation and ROS generation; values are mean ± SEM (*n* = 3). (**B**) and (**C**) Immunoblots of wt/mut p53, BAX and cyt *c*, in cytosolic and mitochondrial fractions of (B) HCT116p53^+/+^ and (C) MDA-MB-231 cells after 16 h with 16 μM SLMP53-1. In B and C, immunoblots are representative of three independent experiments.

In HCT116p53^+/+^ and MDA-MB-231 cells, 16 μM SLMP53-1 led to mitochondrial membrane potential (Δψ_m_) dissipation and reactive oxygen species (ROS) generation (Figure 4A). Consistent with p53-dependence, these effects were not observed in 16 μM SLMP53-1-treated HCT116p53^−/−^ cells (Figure 4A). Additionally, in HCT116p53^+/+^ (Figure 4B) and MDA-MB-231 (Figure 4C) cells, 16 μM SLMP53-1 triggered BAX, wt p53 (Figure 4B) and mut p53R280K (Figure 4C) mitochondrial translocation, and led to cytochrome *c* (cyt *c*) release to cytosol (Figure 4B and 4C). Altogether, these results showed that SLMP53-1 induced a p53-dependent mitochondrial apoptotic pathway in both wt p53- and mut p53R280K-expressing tumor cells.

### SLMP53-1 inhibits the migration of wt p53- and mut p53R280K-expressing tumor cells

**Figure 5:**
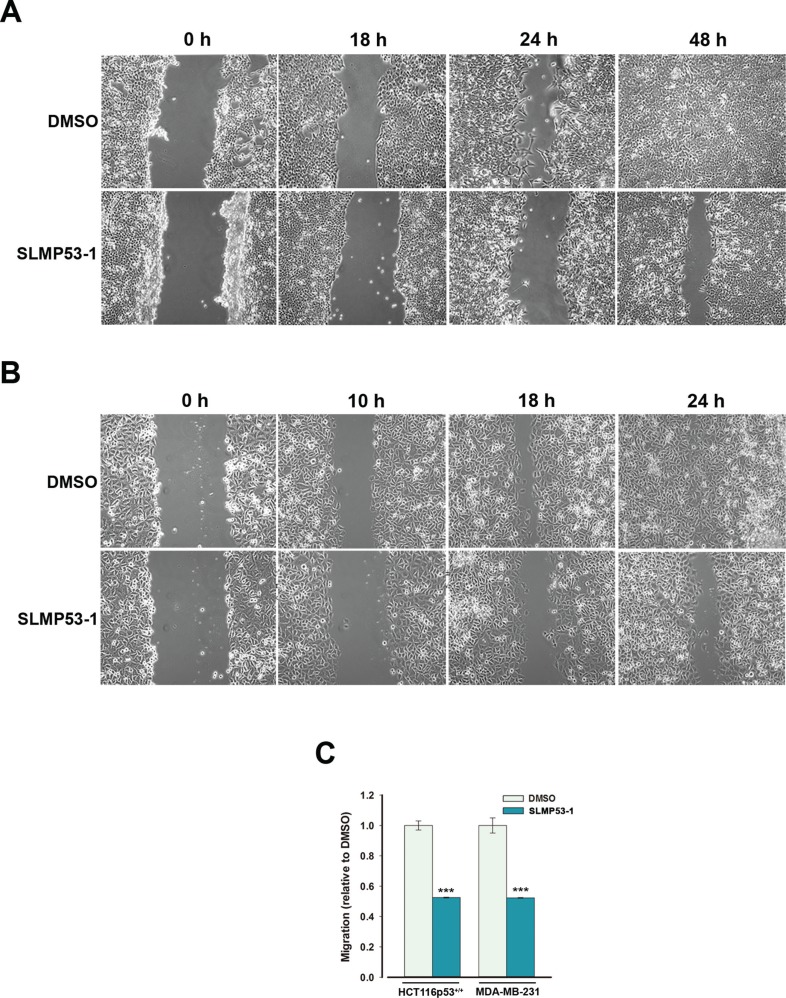
SLMP53-1 prevents the migration of HCT116p53^+/+^ and MDA-MB-231 cells (**A**) HCT116p53^+/+^ and (**B**) MDA-MB-231 confluent cells treated with 4 μM SLMP53-1 (or DMSO only) were observed at different time-points in the wound healing assay. (**C**) Effects of SLMP53-1 on the migration of HCT116p53^+/+^ (7 μM for 24 h) and MDA-MB-231 (16 μM for 8 h) cells analyzed by the chemotaxis assay; the migratory cells were quantified by fluorescence signal, setting as 1 cells treated with DMSO only; data are mean ± SEM (*n* = 3); values significantly different from DMSO only: ***p < 0.001.

The results obtained in the wound healing assay showed that the GI_10_ concentration (4 μM) of SLMP53-1 reduced the migration of HCT116p53^+/+^ and MDA-MB-231 tumor cells (Figure 5A and 5B). These results were corroborated by the chemotaxis cell migration assay, in which the GI_25_ concentration (7 μM) of SLMP53-1 led to over 50% reduction of HCT116p53^+/+^ cell migration compared to DMSO only (Figure 5C). A similar result was obtained with the constitutively more motile MDA-MB-231 cells treated with 16 μM SLMP53-1 (Figure 5C). These data showed that SLMP53-1 inhibited the *in vitro* migration of both wt p53- and mut p53R280K-expressing tumor cells.

### SLMP53-1 sensitizes wt p53- and mut p53R280K-expressing tumor cells to conventional chemotherapeutics in a p53-dependent manner

**Figure 6:**
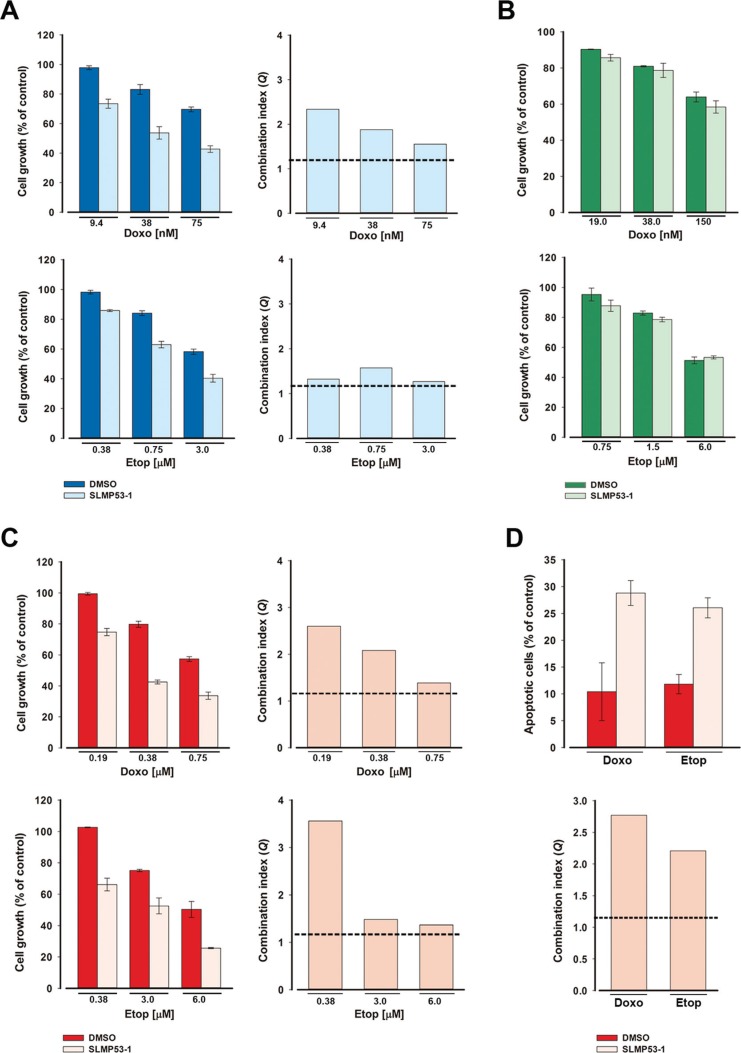
SLMP53-1 sensitizes HCT116p53^+/+^ and MDA-MB-231 cells to the effects of etoposide and doxorubicin (**A**–**C**) Effects of combined treatment with 4 μM SLMP53-1 (or DMSO only) and increasing concentrations of doxorubicin (Doxo) or etoposide (Etop) on tumor cell growth, after 48 h; data are mean ± SEM (*n* = 4); the percentage of cell growth achieved with SLMP53-1 only was (A) 90.3% in HCT116p53^+/+^, (B) 99.8% in HCT116p53^−/−^ and (C) 90.5% in MDA-MB-231 cells. (**D**) Effects of combined treatment with 4 μM SLMP53-1 (or DMSO only) and 0.19 μM doxorubicin or 0.38 μM etoposide on apoptosis after 48 h in MDA-MB-231 cells. The percentage of apoptotic cells obtained with SLMP53-1 only was 0.2%; data are mean ± SEM (*n* = 3). In A, C and D, the *Q* values are represented; dotted line represents *Q* = 1.15.

We investigated whether SLMP53-1 increased the sensitivity of HCT116p53^+/+^ and MDA-MB-231 tumor cells to conventional chemotherapeutics. For that, a low concentration of SLMP53-1, with no significant growth inhibitory effects (GI_10_ of 4 μM), was combined with increasing concentrations of doxorubicin and etoposide. In HCT116p53^+/+^ cells, SLMP53-1 showed synergistic effects with all tested concentrations of chemotherapeutics (*Q* > 1.15), although the most pronounced effects were obtained with doxorubicin (Figure 6A). Conversely, no synergistic effects were observed between SLMP53-1 and the chemotherapeutics in HCT116p53^−/−^ cells (Figure 6B). As in HCT116p53^+/+^ cells, synergistic effects between SLMP53-1 and all tested concentrations of doxorubicin and etoposide were also observed in MDA-MB-231 cells (*Q* > 1.15) (Figure 6C). Interestingly, pronounced synergistic effects between 4 μM SLMP53-1 and the lowest tested concentrations of doxorubicin (0.19 μM) and etoposide (0.38 μM) were also evident regarding total apoptosis in MDA-MB-231 cells (*Q* > 1.15; Figure 6D). Altogether, these results show p53-dependent synergistic effects of SLMP53-1 with conventional chemotherapeutics.

### SLMP53-1 has no *in vitro* toxicity in non-tumorigenic cells

**Figure 7:**
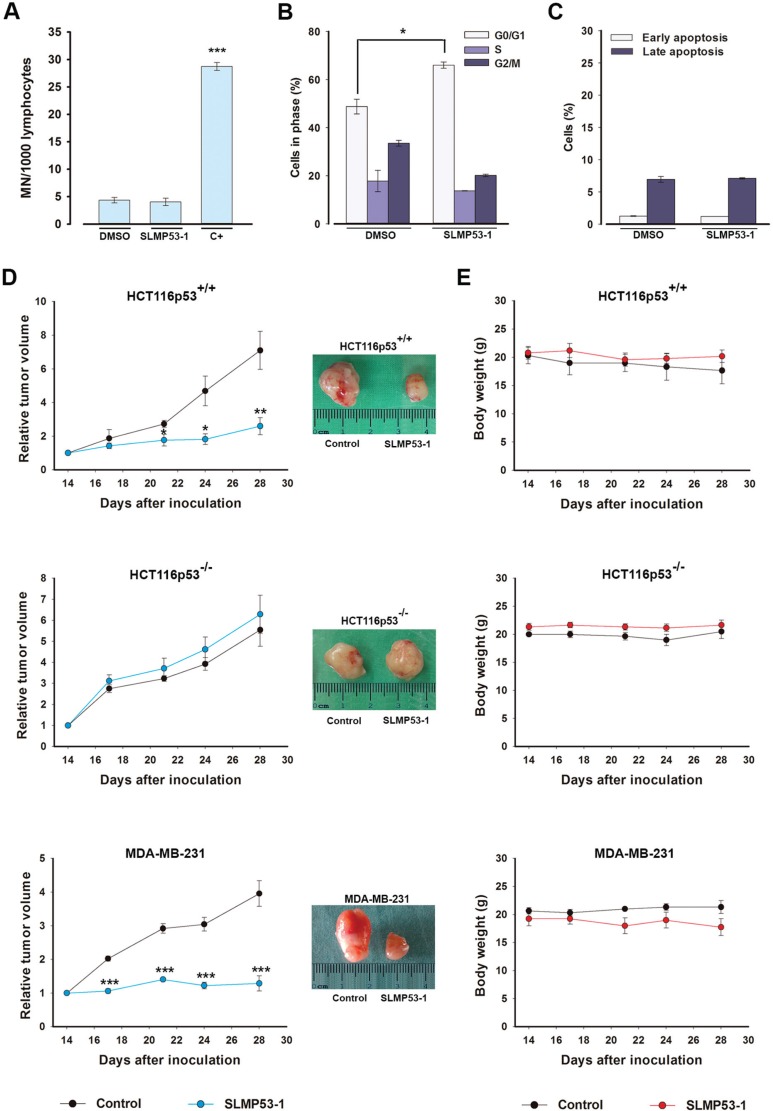
SLMP53-1, with no apparent *in vitro* toxicity, has potent *in vivo* antitumor activity (**A**) Genotoxicity of 16 μM SLMP53-1 by cytokinesis-block micronucleus (MN) assay after 72 h treatment in human lymphocyte cells. Cells treated with 1 μg/mL cyclophosphamide were used as positive control (C+); data are mean ± SEM (*n* = 3). (**B**) Cell cycle after 48 h with 42.4 μM SLMP53-1 in MCF10A cells; data are mean ± SEM (*n* = 3). In A and B, values significantly different from DMSO only: ***p < 0.001. C. Apoptosis after 48 h with 42.4 μM SLMP53-1 in MCF10A cells; data are mean ± SEM (*n* = 3); no significant differences between SLMP53-1 and DMSO only: *p* > 0.05. (**D**) BALB/c nude mice carrying HCT116p53^+/+^, HCT116p53^−/−^ or MDA-MB-231 xenografts treated with 50 mg/kg SLMP53-1 or vehicle (control); values significantly different from control mice: *p < 0.05, **p < 0.01, ***p < 0.001; representative pictures of control and SLMP53-1-treated tumors. (**E**) BALB/c mice body weight during SLMP53-1 treatment; no significant differences between control and SLMP53-1-treated mice: *p* > 0.05.

The potential genotoxicity of SLMP53-1 was investigated using the cytokinesis-block micronucleus assay. At 16 μM, SLMP53-1 did not increase the number of micronuclei in lymphocytes when compared to DMSO only (Figure 7A). Additionally, 16 μM SLMP53-1 induced only 5.6 ± 3.2% (*n* = 5) growth inhibition in non-tumorigenic MCF10A cells, showing that this concentration is not toxic to normal cells. Furthermore, unlike in tumor cells, in MCF10A cells, the growth inhibition induced by SLMP53-1 at GI_50_ concentration (42.4 ± 2.9 μM, *n* = 5) was associated with G0/G1-cell cycle arrest (Figure 7B), but not with apoptosis (Figure 7C).

### SLMP53-1 has potent *in vivo* antitumor activity with no evident toxicity

To evaluate the *in vivo* antitumor potential of SLMP53-1, mice xenograft models carrying HCT116p53^+/+^, HCT116p53^−/−^, or MDA-MB-231 tumor cells were used. The results revealed that four intraperitoneal administrations (twice a week) of 50 mg/kg SLMP53-1 were highly effective, blocking the growth of HCT116p53^+/+^ tumors (Figure 7D) compared to control (vehicle). Most importantly, SLMP53-1 had no effect on HCT116p53^−/−^ tumor xenografts, corroborating its p53-dependent antitumor activity (Figure 7D). Potent antitumor activity was also established for MDA-MB-231 tumors, confirming the *in vivo* efficacy of SLMP53-1 against mut p53R280K-expressing tumors (Figure 7D). No significant loss of body weight or morbidity signs were observed in treated mice compared to control, throughout the experimental period (Figure 7E).

Considering the common chemotherapeutic side effects, some primary toxicity signs potentially triggered by SLMP53-1 were investigated in Wistar rats. For that, the organs relative weight (trophism), as well as biochemical and hematological data were analyzed for three rat groups (saline, vehicle and SLMP53-1) (Supplementary Table 1). No differences between the three groups on relative weight of liver, kidneys, heart and lungs were observed. Concerning biochemical data, only a slight decrease of urea in the vehicle group compared to saline group, and a slight decrease of uric acid and albumin in the SLMP53-1 group compared to vehicle group were observed. Hence, no evident liver or kidney toxicity was detected in SLMP53-1-treated group. Finally, concerning hematological data, just a small increase on the reticulocytes number was observed in the vehicle group compared to saline group, with no alterations between the SLMP53-1 and vehicle groups. Altogether, no apparent toxic side effects were observed for SLMP53-1 on the tissues most commonly affected by conventional chemotherapeutics.

**Figure 8:**
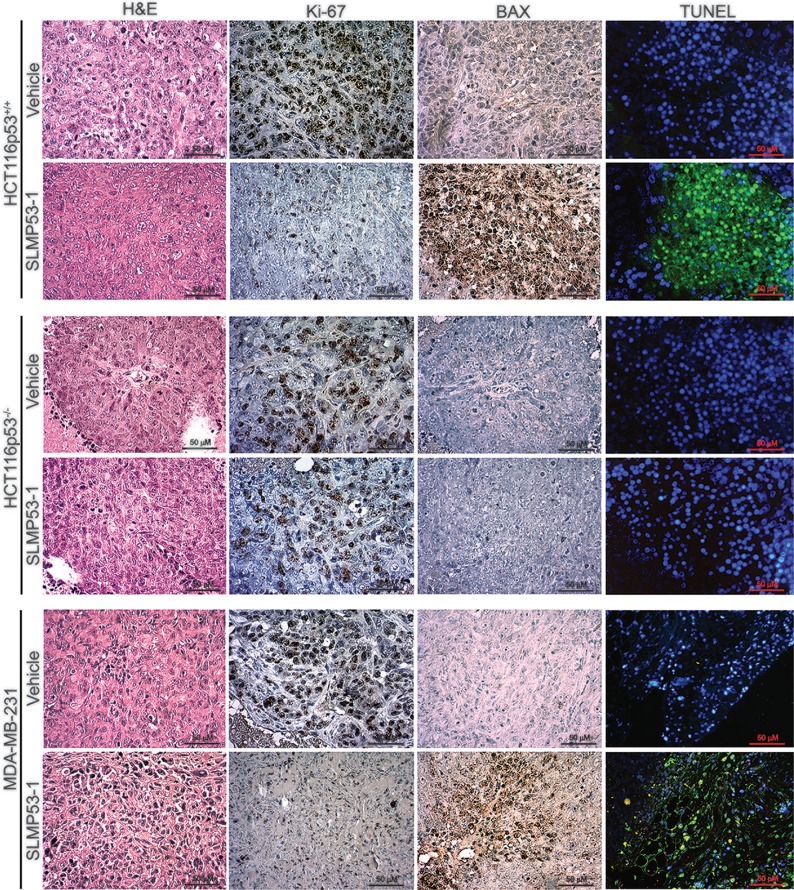
SLMP53-1 decreases cell proliferation and enhances apoptosis in human xenograft tumors Representative images of Ki-67, BAX and DNA fragmentation detection in human HCT116p53^+/+^, HCT116p53^−/−^ and MDA-MB-231 xenograft tumor tissues inoculated in BALB/c nude mice treated with 50 mg/kg SLMP53-1 or vehicle (Scale bar = 50 μm; Magnification 400 ×).

In order to assess the proliferation and apoptotic status of tumors, immunohistochemistry and TUNEL staining were performed for tumor tissues (Figure 8). Both HCT116p53^+/+^ and MDA-MB-231 tumors, treated with SLMP53-1, showed low Ki-67-positive staining (indicative of a low proliferative status), when compared to tumors treated with vehicle (Figure 8). Additionally, a marked increase of BAX expression levels in SLMP53-1-treated tumor tissues, compared to vehicle, was observed (Figure 8). In addition, a pronounced increase of nuclei DNA fragmentation upon SLMP53-1 treatment was observed (Figure 8). On the other hand, a uniform pattern, characterized by high levels of Ki-67 and low levels of BAX expression and DNA fragmentation, between HCT116p53^−/−^ tumors treated with SLMP53-1 and vehicle was obtained (Figure 8).

## DISCUSSION

From the analysis of a chemical library of novel enantiopure tryptophanol-derived oxazoloisoindolinones [16], the small-molecule SLMP53-1 was identified as a new reactivator of wt p53 and mut p53R280K. Considering the relevance of the absolute stereochemical configuration of biologically active compounds, the enantiomer of SLMP53-1 was also synthesized (Figure 1A). Interestingly, this enantiomer was inactive in yeast and exhibited a much lower growth inhibitory effect against wt/mut p53 bearing tumor cells compared to SLMP53-1 (data not shown). These results highlight the relevance of the stereochemistry at positions C-3 and C-9b for the activity of SLMP53-1.

The activation of wt p53 and mut p53R280K (a DNA contact mutant without major structural alterations), associated with the absence of effect on structural mut p53Y220C, lead us to hypothesize some preference of SLMP53-1 for the wt-like conformation of p53. Further studies are underway to analyze the activity of SLMP53-1 on other mut p53s.

Contrary to PRIMA-1, SLMP53-1 showed a p53-dependent tumor growth inhibitory activity. In fact, the induction of cell cycle arrest and apoptosis, and the enhancement of p53 transcriptional activity by SLMP53-1 in wt p53-expressing HCT116 tumor cells were completely abolished in p53-null HCT116 cells. In turn, SLMP53-1 showed a much higher cytotoxicity against mut p53R280K-expressing MDA-MB-231 cells than PRIMA-1 (with previously reported cytotoxicity against this tumor cell line [17]). In these tumor cells, SLMP53-1 restored the wt-like sequence-specific DNA binding ability to mut p53R280K, with subsequent up-regulation of p53 target genes. Curiously, in mut p53R280K-expressing cells, the SLMP53-1-induced growth inhibition was only associated with apoptosis, and not with cell cycle arrest. These results highlight key genetic differences between tumor cells associated with distinct p53 status (wt/mut).

The pathophysiological relevance of the mitochondrial p53 function on apoptotic cell death has been emphasized in several studies [18]. In response to stress, a fraction of p53 translocates to mitochondria, where it binds with Bcl-2 family proteins inducing mitochondrial outer membrane permeabilization (MOMP), cyt *c* release, and subsequent apoptosis [18]. The mitochondrial function of mut p53 is still unclear. Interestingly, mut p53 has been reported to localize constitutively at mitochondrial outer membrane due to its intrinsic cellular stabilization. Despite this, the mut p53 apoptotic activity is compromised by its inability to bind to Bcl-2 and trigger MOMP [18, 19]. In this work, SLMP53-1 triggered a mitochondrial p53 apoptotic pathway in wt p53-expressing tumor cells, involving Δψ_m_ dissipation, p53 and BAX mitochondrial translocation, as well as cyt *c* release. Similar events were triggered by SLMP53-1 in mut p53R280K-expressing tumor cells, supporting the translocation of a functionally active mut p53 to mitochondria.

It is interesting to note that SLMP53-1 treatment induced a p53-dependent ROS generation, which may result from the stimulation of both pro-oxidant and mitochondrial p53 functions. Actually, SLMP53-1 enhances the expression of *BAX* and *PUMA*, which besides a connection with the p53 mitochondrial pathway are also strongly involved in its pro-oxidant function [20].

Collectively, the results obtained indicate that SLMP53-1 is a “double hit” reactivator of p53-mediated apoptosis, stimulating both the transcription-dependent and mitochondrial-mediated p53 functions.

Considered the main cause of morbidity and mortality in cancer patients, tumor cell invasion and metastization are central issues in cancer treatment [21, 22]. The pivotal role of p53 in the prevention of epithelial-mesenchymal transition and migration is widely recognized [22]. Accordingly, mut p53 has been associated to prometastatic functions not only due to the loss of p53 function, but also to the GOF related to p63 inhibition, a well-known suppressor of tumorigenesis and metastasis [22]. Low doses of SLMP53-1 potently inhibited the migration of wt/mut p53-expressing cells, which sustains the reactivation of wt p53, as well as of mut p53 through reestablishment of its wt function and possible inhibition of its GOF.

Drug combination may minimize drug side effects, while increasing tumor suppressor activity [23]. Since the majority of conventional chemotherapeutics, as etoposide and doxorubicin, depend on a functional p53 pathway, combination with p53 reactivators is expected to enhance their efficacy. Indeed, at low doses, SLMP53-1 showed p53-dependent synergistic effects with both chemotherapeutic agents in wt/mut p53-expressing tumor cells. Therefore, combination of SLMP53-1 with chemotherapeutics revealed great therapeutic potential, particularly against multidrug-resistant tumor cells, like triple-negative breast cancers.

The toxicity of standard chemotherapeutics remains a therapeutically limiting problem. Herein, SLMP53-1 was shown to be non-genotoxic. Additionally, despite activating wt p53, at the GI_50_ concentration obtained in tumor cells, SLMP53-1 was not cytotoxic against wt p53-expressing normal MCF10A cells. The minimal cytotoxic effects of p53 reactivators on normal cells have been attributed to their lower susceptibility to apoptotic stimuli when compared to tumor cells with a constitutive activation of a DNA damage signaling pathway [24–26]. This leads normal cells to respond to p53 reactivators with cell cycle arrest (cytostatic effect), instead of cell death (cytotoxic effect) [27, 28]. Actually, this was observed in normal cells with SLMP53-1 at 3 × GI_50_ concentration used in tumor cells. SLMP53-1 can thus be exploited in p53-based cyclotherapy, in which normal cells are protected from the cytotoxicity of chemotherapeutics by induction of cell cycle arrest.

Assays in xenograft mice models reinforced the p53-dependent *in vivo* antitumor activity of SLMP53-1. In fact, SLMP53-1 potently suppressed the growth of wt p53- and mut p53R280K-expressing tumors through inhibition of proliferation and induction of apoptosis, without interfering with the growth of p53-null tumors. Additionally, SLMP53-1 had no apparent toxic side effects on tissues most commonly affected by chemotherapy, such as kidneys, liver and bone marrow.

In conclusion, several evidences are provided in this work supporting the anticancer therapeutic potential of SLMP53-1, both alone and combined with conventional chemotherapeutics. To our knowledge, this is the first report of a pharmacological reactivator of mut p53R280K. Besides its potential applicability in cancer therapy, SLMP53-1 is a starting point for the development of new effective derivatives targeting mut p53s.

## MATERIALS AND METHODS

### Chemical synthesis of SLMP53-1 and its enantiomer

All reagents and solvents were obtained from commercial suppliers and used without further purification. Melting point was determined using a Kofler camera Bock monoscope M. The infrared spectra were collected on a Shimadzu IRAffinity-1 FTIR infrared spectrophotometer. Only noteworthy IR absorptions (cm^−1^) are listed. Microanalyses were performed in a Thermo Scientific TM FLASH 2000 Series CHNS/O analyzer and are within ± 0.5% of theoretical values. Analysis Merck Silica Gel 60 F254 plates were used as analytical thin layer chromatography; flash chromatography was performed on Merck Silica Gel (200–400 mesh). Optical rotations were determined in a Perkin–Elmer 241 MC polarimeter at rt. ^1^H and ^13^C NMR spectra were recorded on a Bruker 400 MHz Ultra-Shield. ^1^H nuclear magnetic resonance spectra were recorded at 400 MHz. Carbon nuclear magnetic resonance spectra were recorded at 100 MHz. ^1^H and ^13^C NMR chemical shifts are reported in δ (ppm) referenced to the solvent used and the proton coupling constants J in hertz (Hz) (Supplementary Figure 1). Spectra were assigned using appropriate COSY, DEPT and HMQC sequences.

Chemical Synthesis of SLMP53-1 (Figure 1A): 2-Acetyl-benzoic acid (0.19 g, 1.16 mmol, 1.1 eq.) was added to a stirred solution of (*S*)-tryptophanol (0.2 g, 1.05 mmol, 1.0 eq.) in 10 mL of toluene. The mixture was heated at reflux under inert atmosphere using Dean-Stark conditions during 16 h. The solvent was removed under reduced pressure and the residue obtained was purified by flash chromatography (Ethyl Acetate/*n*-Hexane 2:1). The product was obtained as a white solid (81% yield) after recrystallization in Ethyl Acetate/*n*-Hexane. SLMP53-1: IR (KBr): 3283 (N-H); 1697 (C = O) cm^−1^; mp: 182 – 184°C; [α]^20^_D_ + 23.7 (*c* 0.43 g/100 ml, CH_2_Cl_2_); ^1^H NMR (DMSO-*d*_6_) δ 10.92 (s, 1H, NH), 7.74 – 7.65 (m, 3H, H-Ar), 7.59 (m, 2H, H-Ar), 7.38 – 7.34 (m, 2H, H-Ar), 7.08 (t, J = 7.1 Hz, 1H, H-Ar), 7.01 (t, J = 7.4 Hz, 1H, H-Ar), 4.41 – 4.29 (m, 2H, CH and OCH_2_), 4.14 (dd, J = 8.0, 6.1 Hz, 1H, OCH_2_), 3.25 (dd, J = 14.5, 5.1 Hz, 1H, CH_2_-indole), 3.12 (dd, J = 14.6, 8.1 Hz, 1H, CH_2_-indole), 1.67 (s, 3H, CH_3_); ^13^C NMR (DMSO-*d*_6_) δ 173.76 (C = O), 147.55 (Cq), 136.63 (Cq), 133.93 (CH-Ar), 131.40 (Cq), 130.81 (CH-Ar), 127.85 (Cq), 124.04 (CH-Ar), 123.87 (CH-Ar), 123.25 (CH-Ar), 121.49 (CH-Ar), 118.86 (CH-Ar), 118.70 (CH-Ar), 111.88 (CH-Ar), 110.60 (Cq), 98.87 (Cq), 74.65 (OCH_2_), 55.95 (CH), 30.84 (CH_2_), 22.79 (CH_3_); Anal. calcd. for C_20_H_18_N_2_O_2_·0.15H_2_O: C 74.81, H 5.67, N 8.73, found: C 74.86, H 5.67, N 8.75.

Chemical Synthesis of SLMP53-1 enantiomer (Figure 1A): Following the same procedure used for the synthesis of SLMP53-1, to a solution of (*R*)-tryptophanol (0.1 g, 0.53 mmol) in toluene (15 mL) was added 2-acetylbenzoic acid (0.10 g, 0.58 mmol). The product was obtained as a white solid (76% yield) after recrystallization in EtOAc/*n*-Hexane. [α]^20^_D_ −27.1 (*c* 0.43 g/100 ml, CH_2_Cl_2_) ^1^H NMR spectra was found to be identical to the one obtained for compound SLMP53-1.

### Plasmids and compounds

The yeast expression vector pLS89-(*TRP1*) encoding human wt p53 under *GAL1–10* inducible promoter (kindly provided by Dr. Richard Iggo; Swiss Institute for Experimental Cancer Research, Switzerland), and pTS76-(*LEU2*) encoding human mut p53R280K or mut p53Y220C under *ADH1* constitutive promoter (kindly provided by Dr. Gilberto Fronza; IST Istituto Nazionale per la Ricerca sul Cancro, Genoa, Italy), were used.

PRIMA-1, doxorubicin and PFT-α were purchased from Sigma-Aldrich, Phikan083 from Tocris and etoposide from Calbiochem. All tested compounds were dissolved in dimethyl sulfoxide (DMSO; Sigma-Aldrich).

### Yeast-based screening assay

*Saccharomyces cerevisiae* (strain CG379) was transformed using the standard lithium acetate method [8]. For expression of human proteins, cells (routinely grown in minimal selective medium) were diluted to 0.05 OD_600_ in induction selective medium containing 2% (w/w) galactose, 1% (w/w) raffinose, 0.7% (w/w) yeast nitrogen base without amino acids from Difco, and a mixture of all the amino acids required for yeast growth (50 μg/mL) except leucine (for pTS76) or tryptophan (for pLS89). Cells transformed with the empty vector (pTS76 or pLS89) were used as control yeast. Yeast cells were incubated at 30°C under continuous orbital shaking (200 r.p.m.), in the presence of 0.1 – 50 μM of compounds or 0.1% DMSO only, for approximately 30 h (time required by control yeast, incubated with DMSO only, to achieve 0.4 OD_600_). In the experiments with PFT-α, yeast cells expressing wt/mut p53 were incubated with 5 μM PFT-α alone and combined with 10 μM SLMP53-1, or DMSO only for approximately 30 h. Yeast growth was analysed by counting the number of colony-forming units (CFU), after 2 days incubation at 30°C, on Sabouraud Dextrose Agar from Liofilchem.

### Yeast cell cycle analysis

Yeast cell cycle progression was analyzed basically as described [29]. Briefly, 1 × 10^7^ cells were fixed in 70% (v/v) ethanol, incubated with 250 μg/mL RNase A (Sigma-Aldrich) and 1 mg/mL Proteinase K (Sigma-Aldrich), followed by 10 μM Sytox Green Nucleic Acid from Invitrogen and flow cytometry analysis.

### Human cell lines and growth conditions

Human colon adenocarcinoma HCT116 cell line expressing wt p53 (HCT116 p53^+/+^) and its isogenic derivative in which full-length p53 has been knocked out (HCT116 p53^−/−^) were kindly provided by Dr. B. Vogelstein (The Johns Hopkins Kimmel Cancer Center, Baltimore, MD, USA); human breast cancer MDA-MB-231, human hepatoma HuH-7, and non-tumorigenic human breast epithelial MCF10A cell lines were from ATCC. All tumor cell lines were routinely cultured in RPMI-1640 medium with ultraglutamine from Lonza supplemented with 10% fetal bovine serum (FBS) from Gibco. MCF10A cell line was cultured in DMEM F12 from Lonza supplemented with MEGM SingleQuot Kit Suppl. & Growth Factors from Lonza. All cell lines were maintained in a humidified incubator at 37°C with 5% CO_2_.

### Sulforhodamine B (SRB) assay

Human cell lines were incubated in 96-well plates, at a final density of 5.0 × 10^3^ cells/well (for HCT116 and HuH-7 cell line), and of 1.0 × 10^4^ cells/well (for MDA-MB-231 and MCF10A cell line), for 24 h. Cells were thereafter treated with serial dilutions of compound (from 1.85 to 150 μM) for 48 h. The effect of compounds on *in vitro* growth was analyzed using the SRB assay as described [10, 30]. The solvent (DMSO; maximum concentration used of 0.25%) was included as control.

For combined treatments, HCT116 p53^+/+^, HCT116 p53^−/−^ and MDA-MB-231 cell lines were treated with the GI_10_ concentration of SLMP53-1 (or DMSO only) and increasing concentrations of doxorubicin (9.38 – 750 nM) or etoposide (0.38 – 6.00 μM). The effect of combined treatments on cell growth was analyzed after 48 h incubation, using the SRB assay, and is expressed by the combination index (*Q*), calculated as reported [30], which indicates: *Q* < 0.85, antagonism; 0.85 < *Q* < 1.15 additive effect; *Q* > 1.15, synergy.

### Analysis of cell cycle and apoptosis in human cell lines

HCT116, MDA-MB-231 and MCF10A cell lines were incubated in 6-well plates, at a final density of 1.5 × 10^5^ cells/well (for HCT116 cell line) and 2.3 × 10^5^ cells/well (for MDA-MB-231 and MCF10A cell lines), for 24 h. Cells were thereafter treated with SLMP53-1 or DMSO only for 24 h. For cell cycle analysis, cells were stained with propidium iodide (PI; Sigma-Aldrich) followed by flow cytometric analysis, as described [30]. For apoptosis analysis, cells were analyzed by flow cytometry using the Annexin V-FITC Apoptosis Detection Kit I from BD Biosciences according to the manufacturer's instructions; synergistic effects of combined treatments between the GI_10_ concentration of SLMP53-1 (or DMSO only) and 0.19 μM doxorubicin or 0.38 μM etoposide, in MDA-MB-231 cell lines, were analyzed (determination of *Q* value) as described in the SRB assay.

### Western blot analysis

Whole protein extracts (from yeast and human cells), and mitochondrial/cytosolic fractions from human cell lines were prepared as described [30]. Western blot analysis was performed as described [30] using a mouse monoclonal anti-p53 (DO-1), anti-MDM2 (SMP14), anti-BAX (2D2), anti-PUMA (B-6), anti-PARP (C2–10), and anti-cytochrome *c* (A8) antibodies followed by an anti-mouse horseradish-peroxidase (HRP)-conjugated secondary antibody. For p21 detection, membranes were probed with a rabbit polyclonal anti-p21 (C–19) antibody, followed by an anti-rabbit HRP-conjugated secondary antibody. For loading control, membranes were stripped and reprobed with a mouse monoclonal anti-yeast phosphoglycerate kinase (Pgk1p) or anti-GAPDH (6C5) antibodies. For analysis of mitochondrial and cytosolic fractions, membranes were reprobed with the loading controls mouse monoclonal anti-COX IV (F-8) and anti-GAPDH (6C5) antibodies, respectively. With the exception of anti-Pgk1p (Alfagene), all antibodies were purchased from Santa Cruz Biotechnology. Western blots presented are representative of three independent experiments.

### Dual-luciferase reporter assay in human tumor cell lines

Dual-luciferase reporter assay was performed basically as reported [29, 31]. Briefly, about 5 × 10^4^ cells/well of HCT116 tumor cell line were incubated in 24-well plates for 24 h. Cells were thereafter transfected at approximately 80% confluence using the Myrus LT-1 reagent, and according to the manufacturer's instructions. Specifically, 350 ng of the reporter vectors containing either the p21 (pGL3-1138) or the MDM2 (pGL3-MDM2) promoter were used along with 50 ng of the control pRLSV40 plasmid introduced to normalize the transfection efficiency. After transfection, cells were treated with the GI_50_ and 2 × GI_50_ concentration of SLMP53-1 (or DMSO only), for 16 h. Cells were harvested, and the luciferase assay was carried out using the dual-luciferase reagent. The p53 transcriptional activity is directly proportional to the luciferase activity (measured by the emitted light intensity) as described [29].

### RNA extraction and quantitative RT-PCR (qPCR)

Total RNA from tumor cell lines was extracted using the RNeasy Kit (Qiagen). About 1 μg of RNA was used for cDNA synthesis using M-MuLV reverse transcritptase and RevertAid cDNA Synthesis kit (ThermoFisher) in 20 μL final volume, and following manufacturer's instructions. qPCR assays were performed in a 384-well plate format on a CFX Touch Real-Time PCR Detection System (Bio-rad), starting with 25 ng of cDNA, as previously described [32]. The 2X KAPA SYBR^®^ FAST qPCR Kit (Kapa Biosystems) and specific primers, purchased from Eurofins (MWG), were employed. *GAPDH, B2M* and *ACTB* were used as reference genes.

### TransAM assay

About 1.5 × 10^6^ cells of MDA-MB-231 cell line were incubated in T-75 flasks for 24 h. Cells were thereafter treated with 2 × GI_50_ and 3 × GI_50_ concentration of SLMP53-1 (or DMSO only) for 24 h. Nuclear protein extracts were obtained using the Nuclear Extract Kit from Active Motif, and the protein concentration was measured using NanoDrop 1000 from Thermo scientific. The p53 DNA binding ability was evaluated using the TransAM p53 Transcription Factor Assay Kit from Active Motif, according to the manufacturer's instructions. Briefly, equal amount of total nuclear protein was loaded into a 96-well plate coated with an immobilized oligonucleotide containing a p53 consensus binding site. The amount of bound p53 protein was quantified using an anti-p53 antibody followed by a HRP-conjugated secondary antibody. The HRP signal was measured after addition of a manufacturer's substrate, using the Bio-Tek Synergy HT plate reader at 450 nm. A nuclear extract of wt p53-expressing MCF-7 cells treated with H_2_O_2_ was used as kit positive control.

### Transfection of p53 siRNA

MDA-MB-231 cells were transfected with 100 nM of small interfering RNAs (siRNAs) against p53 (SMARTpool p53) and negative nonspecific siRNAs (Non-targeting Pool) from Thermo Scientific/Dharmacon using Lipofectamine 2000 (Invitrogen), following the manufacturer's instructions. After 24 h transfection, cells were treated with GI_50_ concentration of SLMP53-1 (or DMSO only) for 24 h.

### Analysis of Δψ_m_ dissipation and ROS generation

Analyses of Δψ_m_ dissipation and ROS generation were performed as previously reported [30]. Briefly, about 1.5 × 10^5^ cells/well (for HCT116 cell line) and 2.3 × 10^5^ cells/well (for MDA-MB-231 cell line) were incubated in 6-well plates for 24 h. Cells were thereafter treated with the GI_50_ concentration of SLMP53-1 (or DMSO only) for 8 h (for HCT116 cell line) or 16 h (for MDA-MB-231 cell line) in Δψ_m_ analysis, and for 24 h in ROS analysis. Then, cells were stained with 5 μM Cell-ROX Green Reagent from Life Technologies (in ROS analysis) or with 1 nM DiOC_6_(3) from Alfagene (in Δψ_m_ analysis) for 30 min at 37°C; followed by flow cytometry analysis.

### *In vitro* migration assays

Cell migration was analyzed using the Wound Healing Scratch assay (as described in [33]) and the QCM 24-Well Fluorimetric Chemotaxis Cell Migration Kit (8 μm), from Merck Millipore. In the Wound Healing Scratch assay, about 5 × 10^5^ cells/well of HCT116 p53^+/+^ and MDA-MB-231 cell lines were grown to confluence in 6-well plates for 24 h. Using a sterile 200 μL tip, a fixed-width wound was created in the cell monolayer and the GI_10_ concentration of SLMP53-1 (or DMSO only) was added to medium. Cells were thereafter photographed using the Moticam 5.0MP camera with Motic's AE2000 inverted microscope with 400x magnification at different time-points of treatment until complete closure of the wound. In the QCM 24-Well Fluorimetric Chemotaxis Cell Migration Kit (8 μm), about 0.5 × 10^6^ cells/mL of HCT116 p53^+/+^ and MDA-MB-231 cell lines were prepared in serum free RPMI 1640 treated with the GI_25_ (for HCT116 p53^+/+^ cell line) or GI_50_ (for MDA-MB-231 cell line) of SLMP53-1 (or DMSO only), and incubated for 24 h or 8 h, respectively. The prepared cell suspensions were distributed in 24-well plates (300 μL/insert), followed by addition of 500 μL medium containing 10% FBS to the lower chamber. Cells that migrated through the 8 μm pore membranes were eluted, lysed and stained with a green-fluorescence dye that binds to cellular nucleic acids. The number of migrated cells is proportional to the fluorescence signal measured using the Bio-Tek Synergy HT plate reader at 480/520 nm (ex/em).

### Genotoxicity studies by micronucleus assay

Genotoxicity was analyzed by cytokinesis-block micronucleus assay in lymphocytes as described [34]. Fresh peripheral blood samples were collected from healthy volunteers into heparinized vacutainers. Blood samples, suspended in RPMI medium supplemented with 10% FBS, were treated with the GI_50_ concentration of SLMP53-1, DMSO only or 1 μg/mL cyclophosphamide (known mutagenic agent; positive control; Sigma-Aldrich) for 44 h. Cells were thereafter treated with 3 μg/mL cytochalasin B (cytokinesis preventive; Sigma-Aldrich) for 28 h. Lymphocytes were isolated by density gradient separation (Histopaque-1077 and -1119; Sigma-Aldrich,), fixed in 3:1 methanol/glacial acetic acid, and stained with Wright stain (Sigma-Aldrich). For each sample, 1000 binucleated lymphocytes were blindly scored using a Leica light optical microscope (Wetzlar); the number of micronuclei per 1000 binucleated lymphocytes was recorded.

### *In vivo* antitumor and toxicity assays

Animal experiments were conducted according to the European Council Directives on Animal Care and to the National Authorities. The BALB/c Nude mice and Wistar rats were purchased from Charles-River Laboratories and housed under pathogen free conditions in individual ventilated cages. For toxicity assays, Wistar rats were treated with 50 mg/kg SLMP53-1, vehicle (DMSO) or saline solution (control) by intraperitoneal injection, twice a week during two weeks. After the four administrations, samples of blood and organs (kidneys, liver, heart and lungs) were collected for toxicological analysis. Each group was composed of five animals. Xenograft tumor assays were performed with HCT116p53^+/+^, HCT116p53^−/−^ and MDA-MB-231 human tumor cell lines. Briefly, 1 × 10^6^ HCT116 cells (in PBS) or 5 × 10^6^ MDA-MB-231 cells (in PBS/BD Matrigel Matrix High Concentration, 1:1; BD Biosciences) were inoculated subcutaneously in the dorsal flank of each mice. Tumor dimensions were assessed by caliper measurement and their volumes were calculated [tumor volume = (length × width^2^) / 2]. Treatment was started when tumors reached volumes of approximately 100 mm^3^ (which occurred 14 days after the grafts). Mice were then treated twice a week with 50 mg/kg SLMP53-1 or vehicle (control) by intraperitoneal injection during two weeks. Tumor volumes and body weights were monitored twice a week until the end of the treatment. Animals were sacrificed by cervical dislocation at the end of the study. After sacrifice, tumors were removed and photographed. Human endpoints were established namely euthanasia in case of tumors reached 1500mm3, or if the animals presented any signs of morbidity. The following number of animals was used: HCT116p53^+/+^ and HCT116p53^−/−^ cell lines (Control-6, Treatment-6), MDA-MB-231 (Control-6, Treatment-6).

### Immunohistochemistry

Tumor tissues were fixed in 10% formalin, embedded in paraffin, sectioned at 4 μm, and stained with hematoxylin and eosin (H & E) or antibodies, following standard methodologies. Briefly, after deparaffination and rehydration, antigen retrieval was performed by boiling the sections for 20 min in 10 mM citrate buffer (pH 6.0). The slices were then held for 20 min at room temperature. After dewaxing and blocking the endogenous peroxidases with UltraVision Hydrogen Peroxide Block, sections were treated with UltraVision Protein Block solution, both from Lab Vision Thermo Scientific. Incubation with primary anti-Ki-67 (SP6) and anti-BAX (6A7) antibodies, from Pierce Thermo Scientific, was performed for 2 h, at room temperature. Immunostaining was carried out using the UltraVision Quanto Detection System HRP DAB kit, from Lab Vision Thermo Scientific, according to the manufacturer's instructions. Tissue sections were counterstained with Gill's hematoxylin from Thermo Scientific, dehydrated, clarified and mounted. The primary antibody was replaced by 10% non-immune serum for negative controls. Finally, images were obtained using the Motic's AE2000 inverted microscope (400 × magnification) with the Moticam 5.0 MP camera.

### TUNEL assay

TUNEL assay was performed using the *in situ* Cell Death Detection Kit Fluorescein from Roche, according to the manufacturer's instructions. Briefly, after deparaffination and rehydration, tissues sections were treated for 20 min in 10 mM citrate buffer (pH 6.0) and incubated with TUNEL Reaction Mixture for 60 min at 37^°^C in the dark. Tissues were counterstained with DAPI (0.1 μg/mL). Images were obtained using an Eclipse E400 fluorescence microscope (Nikon), 400 × magnification, with a Digital Sight camera system (Nikon DS-5Mc), carrying built-in software for image acquisition (Nikon ACT-2U).

### Flow cytometric data acquisition and analysis

For the flow cytometric analysis, the AccuriTM C6 flow cytometer from BD Biosciences and the CellQuest software from BD Biosciences were used. For the identification and quantification of cell cycle phases the FlowJo software was used.

### Statistical analysis

Data were analyzed statistically using the GraphPad software. Differences between means were tested for significance using the Student's *t*-test (*p < 0.05; **p < 0.01; ***p < 0.001).

## SUPPLEMENTARY MATERIAL TABLE AND FIGURE


