# Supplementary material for: Reactivation of wild-type and mutant p53 by tryptophanolderived oxazoloisoindolinone SLMP53-1, a novel anticancer small-molecule
Source: Oncotarget. 2015 Dec 28;7(4):4326–43. doi: 10.18632/oncotarget.6775 (PMC4826208; doi:10.18632/oncotarget.6775)
Supplement: Supplementary file 1 [file oncotarget-07-4326-s001.pdf]

Reactivation of wild-type and mutant p53 by tryptophanol-derived oxazoloisoindolinone SLMP53-1, a novel anticancer small-molecule

Supplementary Materials

Supplementary Table S1: Safety profile for the three mice groups under study at the final time point

	Saline	Vehicle	Treated
Body weight and relative tissue weight (trophism)			
BW (g)	340.30 ± 13.45	361.80 ± 0.01	348.30 ± 0.01
Heart/BW (g/kg)	3.03 ± 0.03	3.01 ± 0.09	2.91 ± 0.07
Liver/BW (g/kg)	38.71 ± 2.36	38.52 ± 0.89	35.74 ± 0.81
Kidney/BW (g/kg)	7.08 ± 0.40	6.81 ± 0.27	6.55 ± 0.34
Lung/BW (g/kg)	3.89 ± 0.07	3.81 ± 0.08	3.83 ± 0.11
Biochemical data			
Blood Glucose (mg/dL)	188.0 ± 7.51	207.20 ± 7.00	186.80 ± 11.12
Urea (mg/dL)	20.67 ± 0.77	18.34 ± 0.24*	18.48 ± 1.31
Uric acid (mg/dL)	1.07 ± 0.07	0.82 ± 0.10	0.78 ± 0.05*
Creatinine (mg/dL)	0.30 ± 0.00	0.30 ± 0.01	0.30 ± 0.01
Total Proteins (g/dL)	5.50 ± 0.00	5.35 ± 0.17	5.08 ± 0.15
Albumin (g/dL)	3.03 ± 0.03	2.90 ± 0.05	2.73 ± 0.09*
ALT (U/L)	36.00 ± 2.89	30.75 ± 3.30	32.50 ± 4.87
AST (U/L)	95.33 ± 25.36	56.25 ± 4.60	67.00 ± 11.05
CK (U/L)	458.30 ± 148.60	229.50 ± 64.40	281.80 ± 101.90
Total Chol (mg/dL)	44.33 ± 1.76	52.40 ± 3.54	60.25 ± 5.98
Triglycerides (mg/dL)	107.00 ± 16.29	161.40 ± 17.55	169.80 ± 24.00
Hematological data			
RBC count ($x10^6/\mu L$)	7.10 ± 0.15	7.41 ± 0.28	7.28 ± 0.27
HGB (g/dL)	13.97 ± 0.18	13.95 ± 0.46	13.53 ± 0.25
HCT (%)	40.50 ± 0.82	42.28 ± 2.03	39.63 ± 0.99
WBC counts ($x10^3/\mu L$)	5.23 ± 0.52	5.95 ± 1.61	5.08 ± 0.434
PLT counts ($x10^3/\mu L$)	811.00 ± 29.61	793.80 ± 28.35	852.50 ± 32.05
PCT (%)	0.47 ± 0.03	0.46 ± 0.009	0.50 ± 0.02
RET counts (%)	2.80 ± 0.12	3.56 ± 0.23*	3.28 ± 0.23

The results are expressed as mean ± SEM (n = 4–6). *$p < 0.05$ versus Control group; ALT, alanine aminotransferase; AST, aspartate aminotransferase; BW, body weight; CK, Creatine Kinase; HCT, hematocrit; HGB, Hemoglobin concentration; PCT, plateletcrit; PLT, platelet; RBC, red blood cell count; RET, reticulocytes; WBC, white blood cell.

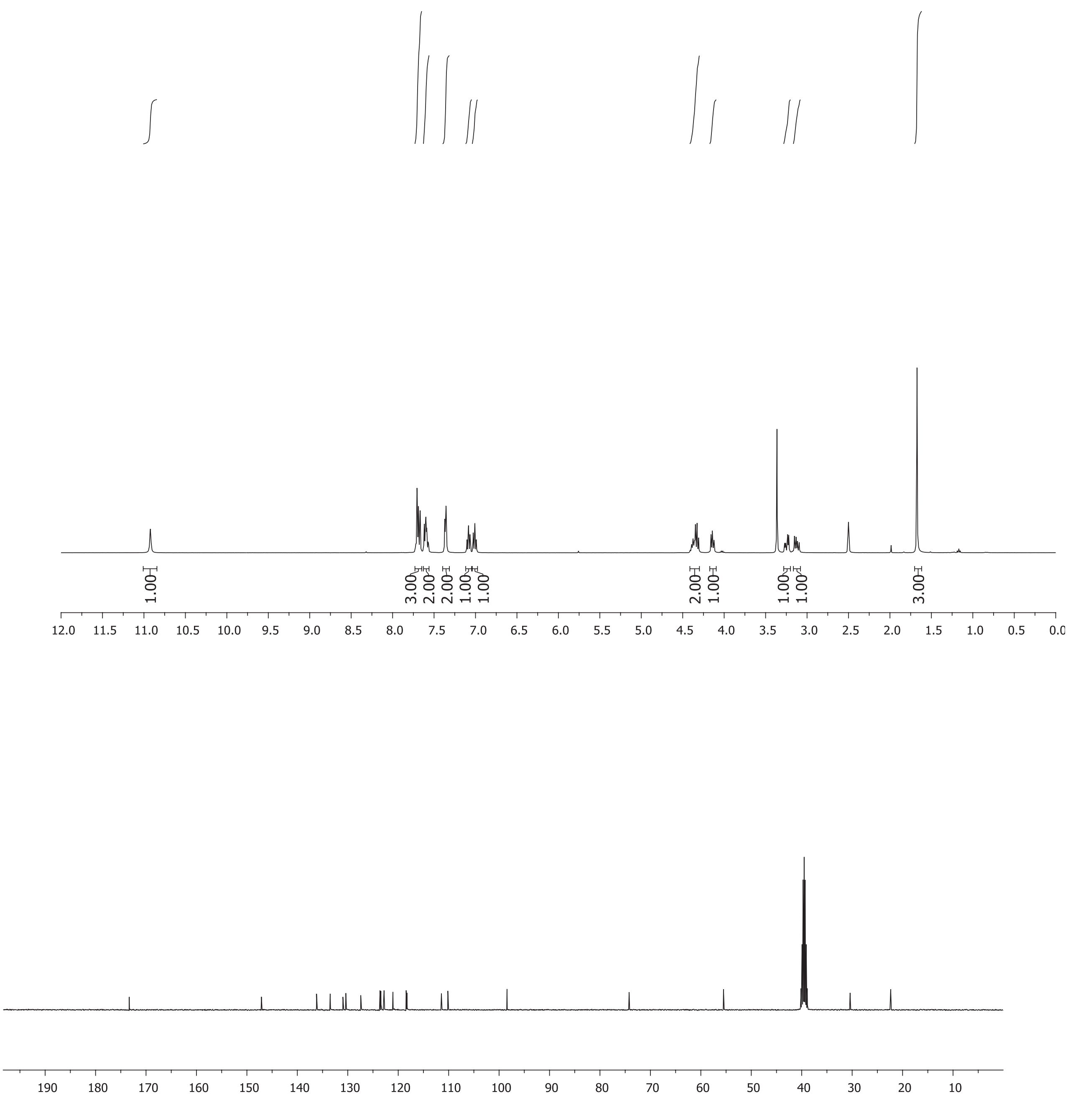

Supplementary Figure S1: ^{1}H NMR and ^{13}C NMR data for compound SLMP53-1.
